# Fluctuations in dietary crude protein content affect rumen bacterial community and metabolome in Holstein dairy cows

**DOI:** 10.3389/fvets.2025.1645129

**Published:** 2025-09-02

**Authors:** Chuankai Zhang, Yifan Liu, Jiaying Wang, Landan Xing, Lei Liu, Yingkai Quan, Liukuan He, Liyang Zhang, Tengyun Gao, Tong Fu, Kaizhen Liu, Hongxia Lian

**Affiliations:** College of Animal Science and Technology, Henan International Joint Laboratory of Nutrition Regulation and Ecological Raising of Domestic Animals, Henan Agricultural University, Zhengzhou, China

**Keywords:** dietary crude protein, fluctuations, bacterial community, metabolomics, nitrogen, Holstein

## Abstract

**Introduction:**

To elucidate the effects of fluctuating dietary crude protein (CP) on Holstein dairy cows, this study investigated lactation performance, rumen bacterial communities, and metabolome profiles.

**Methods:**

In a 60-day trial, twenty-four Holstein cows were randomly assigned to a fluctuating CP diet (FCP), high CP diet (HCP, 18.05% CP), or intermediate CP diet (ICP, 16.05% CP). FCP alternated between high (18.05%, FCP-HCP) and low CP (14.04%, FCP-LCP) every 48 h, forming a 96-h cycle. During the final cycle, blood, milk, and urine samples were collected to measure plasma urea nitrogen (PUN), milk urea nitrogen (MUN), and urine urea nitrogen (UUN). Rumen bacterial diversity and metabolomic profiles were also analyzed.

**Results:**

Fluctuating CP did not significantly affect lactation performance (*p* > 0.05). PUN in FCP was lower than HCP on Day 1 (*p* < 0.01), while MUN in ICP was lower than HCP and FCP on Day 2 (*p* < 0.01). On Day 3, PUN and UUN in FCP exceeded HCP and ICP (*p* < 0.05), and on Day 4, PUN in FCP was again lower than HCP (*p* < 0.01). FCP had lower Proteobacteria on Day 2 (*p* < 0.05) and lower Patescibacteria on Days 3–4 (*p* < 0.05). Metabolomics indicated shifts mainly in amino acid, energy, purine, and pyrimidine metabolism.

**Discussion:**

These findings show that short-term CP fluctuations induce dynamic changes in nitrogen excretion, rumen bacterial composition, and key metabolic pathways without affecting milk production. This suggests that dietary CP variation primarily influences nitrogen metabolism and rumen bacterial dynamics rather than lactation performance.

## 1 Introduction

Dietary crude protein (CP) is the primary source of nitrogen for dairy cows. However, nitrogen use efficiency (NUE) remains low due to metabolic and digestive constraints. When nitrogen intake exceeds physiological demands, the surplus is mainly excreted as urinary urea, while undigested protein contributes to fecal nitrogen loss, leading to substantial nitrogen excretion (1). For instance, lactating dairy cows producing approximately 9,500 kg of milk annually excrete about 0.9 to 1.1 kg of nitrogen per day via combined fecal and urinary routes (2). Urinary nitrogen is the primary contributor to nitrogen volatilization, even though urinary and fecal nitrogen contribute nearly equally to total nitrogen excretion in lactating dairy cows (3). Therefore, developing feeding strategies that reduce nitrogen excretion without compromising production performance is crucial for enhancing nitrogen use efficiency and minimizing the environmental impact of dairy farming.

Historically, in the pursuit of higher milk yields, dairy cows in many countries were fed diets with excessively high CP, often resulting in suboptimal NUE (4). The inverse relationship between dietary CP levels and NUE is primarily due to the inefficient conversion of degraded dietary protein into microbial protein. This inefficiency becomes especially pronounced during periods of rapid CP degradation, when ruminal protein breakdown proceeds too quickly and produces excess ammonia. Without adequate fermentable energy to synchronize with nitrogen release, excess ammonia cannot be efficiently utilized for microbial protein synthesis, thereby limiting microbial growth and overall nitrogen utilization (5). Given the well-established linear correlation between nitrogen intake and excretion, strategically lowering dietary CP levels is widely recognized as a key strategy to mitigate nitrogen waste and improve NUE (6). Improving NUE is essential not only for reducing environmental nitrogen pollution, but also as a key indicator of feeding management efficiency in modern dairy systems, aligning sustainability with productivity goals (7).

To maintain dairy cow productivity while reducing dietary crude protein (CP) levels, fluctuating protein supply strategies have garnered increasing attention. For example, calves fed diets alternating between high and low CP every 48 h showed significantly improved nitrogen retention (8). Similarly, cyclic CP diets effectively reduced total nitrogen requirements in finishing beef cattle (9). In lactating dairy cows, studies suggest that fluctuating CP supply can enhance NUE, possibly through increased gastrointestinal absorption of recycled urea nitrogen. Moreover, rumen microbial communities exhibit high adaptability to changes in nitrogen availability, dynamically modulating nitrogen metabolism to buffer temporal imbalances between degradable nitrogen and fermentable organic matter (10, 11). However, despite these promising observations, the effects of fluctuating CP supply in lactating cows remain inconsistent. Erickson et al. found that this strategy did not improve nitrogen retention but instead reduced nitrogen digestibility and shifted nitrogen excretion from urine to feces (12). These discrepancies highlight the need for further research to elucidate the specific mechanisms and outcomes of CP fluctuation strategies in dairy cow nutrition.

To explore how fluctuations in dietary CP levels affect nitrogen metabolism in dairy cows, this study investigated the effects of a cyclic CP feeding strategy on rumen bacterial communities and their functional roles. We hypothesized that alternating high and low CP levels would induce shifts in rumen bacterial composition and metabolic pathways, thereby stabilizing nitrogen metabolism in response to dietary nitrogen fluctuations. To test this hypothesis, we compared a fluctuating CP diet (FCP) with constant high (HCP) and intermediate (ICP) CP diets, allowing us to isolate the effects of CP fluctuation from total protein intake. This experimental design aimed to capture transient bacterial and metabolic responses, offering new insights into how short-term dietary protein variation affects nitrogen utilization in dairy cows.

## 2 Materials and methods

### 2.1 Animal care

The experiment was conducted at the experimental farm of Henan Agricultural University (Zhengzhou City, Henan Province) and was approved by the Animal Care Committee of Henan Agricultural University (Permit Number: 12–1,328).

### 2.2 Animals, experimental design, and diets

A completely randomized design was used to assign 24 Holstein cows (average body weight: 569.23 ± 47.12 kg; days in milk: 150.32 ± 19.10 d; milk yield: 22.23 ± 2.80 kg/d) into three treatment groups (*n* = 8 per group). The dietary treatments included a Fluctuating CP Diet (FCP), a High CP Diet (HCP, 18.05% CP), and an Intermediate CP Diet (ICP, 16.05% CP). The FCP group alternated between a high CP diet (18.05% CP, FCP-HCP) and a low CP diet (14.04% CP, FCP-LCP) every 48 h, completing one full cycle every 96 h (Table 1). The formal experimental period lasted 60 days, during which the FCP group underwent 15 complete fluctuation cycles, each comprising 48 h of high CP diet followed by 48 h of low CP diet. A 16-day pre-experimental adaptation period was provided.

**Table 1:** Experimental design and dietary treatments.

Item	HCP group (*n* = 8)	ICP group (*n* = 8)	FCP group (*n* = 8)
Dietary strategy	Static high CP	Static intermediate CP	Cyclic CP fluctuation
CP levels (DM basis, %)	18.05%	16.05%	18.05% (48 h, High-CP phase) 14.04% (48 h, Low-CP phase)
Total duration	60 days	60 days	60 days (15 complete cycles)

All cows were individually housed in 4 meters × 5 meters stalls within a portal-frame building with a concrete floor and tin roof. They were fed the experimental diets as a total mixed ration (TMR) twice daily (7:00 and 15:00) and milked three times daily (06:00, 12:00, and 18:00). Feed samples were collected before the start of the experimental period and dried in a laboratory oven at 60 °C for 24 h. The samples were then further dried at 105°C to a constant weight, ground using a mill, and sieved through a 1 mm mesh for further analysis. Crude protein (CP), dry matter (DM), crude ash (Ash), ether extract (EE), calcium (Ca), and phosphorus (P) contents were determined according to AOAC methods 990.03, 930.15, 942.05, 920.39, 968.08, and 965.17, respectively (13). Neutral detergent fiber (NDF) and acid detergent fiber (ADF) contents were analyzed according to Van Soest et al. (14) using an Ankom Fiber Analyzer (Ankom Technology, Fairport, NY, United States). The chemical and nutritional composition of the basal diet is detailed in Table 2. Except for CP levels, other nutrients were kept as consistent as possible across all diets. Daily feed intake was recorded and weighed for each cow, and the average daily intake was calculated. Cows had free access to water and movement, with water supplied via individual drinking sinks.

**Table 2:** The ingredients and compositions of the diets (% DM basis).

Items	Content (% DM)			
Ingredient	HCP	ICP	FCP-HCP	FCP-LCP
Corn	23.51	27.96	23.51	31.49
Distillers Dried Grains with Soluble	6.53	7.74	6.53	11.13
Soybean meal	12.09	7.88	12.09	1.45
Cottonseed meal	2.90	1.45	2.90	0.97
CaHPO_4_	0.48	0.48	0.48	0.48
Limestone	0.97	0.97	0.97	0.97
Salt	0.48	0.48	0.48	0.48
Premix^a^	0.53	0.53	0.53	0.53
Sodium bicarbonate	0.73	0.73	0.73	0.73
Magnesium oxide	0.15	0.15	0.15	0.15
Molasses	3.29	3.29	3.29	3.29
Maize silage	18.09	18.09	18.09	18.09
Soybean hull	6.13	6.13	6.13	6.13
Cottonseed	4.03	4.03	4.03	4.03
Alfalfa hay	14.83	14.83	14.83	14.83
Peanut hay	5.24	5.24	5.24	5.24
Total	100.00	100.00	100.00	100.00
Nutrient levels (%)				
Dry matter	79.41	79.33	79.41	79.30
NE_L_^b^(MJ/kg)	6.61	6.61	6.61	6.61
Crude protein	18.05	16.05	18.05	14.04
Ether extract	3.02	2.97	3.02	3.19
Neutral detergent fiber	29.10	28.71	29.10	28.79
Acid detergent fiber	17.28	16.78	17.28	16.37
Ash	9.12	8.11	9.12	7.93
Ca	1.06	1.05	1.06	1.04
P	0.42	0.39	0.42	0.37

FCP-HCP: Stage with high CP content in the treatment group fluctuating CP diet; FCP-LCP: Stages with low CP content in the treatment group fluctuating CP diet; ICP: intermediate CP.

a The premix provided the following per kg of diets: vitamin A, 150,000 IU; vitamin D3, 35,000 IU; vitamin E, 3000 IU; niacinamide, 2,000 mg; biotin, 200 mg; β-carotene, 300 mg; manganese, 1,500 mg; copper, 650 mg; zinc, 2,800 mg; iodine, 35 mg; cobalt, 23 mg; and selenium, 25 mg.

b Except for the NE_L_, which is a calculated value, all others are measured values, calculated according to the NRC (2001) model.

### 2.3 Sample collection and measurements

During the experimental period, daily milk yield was recorded for each cow, and milk samples were collected on days 9–12 (3rd cycle), 25–28 (7th cycle), 40–44 (11th cycle), and 53–56 (14th cycle) of the trial. Milk samples collected from the morning, midday, and evening milkings were pooled in a 4:3:3 ratio and then divided into two portions for analysis. One portion was treated with 0.06% potassium dichromate and stored at 4 °C, while the other was frozen at −20 °C without any preservative. Both sets of samples were transported to the Henan Dairy Production Performance Measurement Center in Zhengzhou for the analysis of milk composition and milk urea nitrogen (MUN) concentrations.

From days 53 to 56 of the experiment (14th cycle), 10 mL midstream urine samples were collected every 6 h from each cow via vulvar stimulation, and the samples collected on the same day were pooled. The pooled samples were acidified by slowly adding 1 mL of concentrated sulfuric acid (98%) to every 10 mL of urine to prevent nitrogen loss and sample degradation, and then stored at −20 °C (15). Before analysis, all urine samples were thawed and appropriately diluted. Similarly, daily blood samples were collected from the coccygeal vein of each cow before the morning feeding, while the cows were fasted. Whole blood samples were centrifuged at 3,000 × g for 15 min at 4 °C to separate the plasma, which was then stored at −20 °C until analysis. Urine and plasma urea concentrations were determined using a commercial urea assay kit (Abcam, ab83362), and the concentrations of urine urea nitrogen (UUN) and plasma urea nitrogen (PUN) were calculated based on these measurements.

From days 53 to 56 of the experiment (14th cycle), rumen fluid samples were collected daily from each cow using an esophageal tube 2 h after feeding, a time chosen to capture peak fermentation activity. The first 50 mL of fluid was discarded to minimize saliva contamination, and approximately 100 mL of rumen fluid was then collected. The rumen fluid was immediately filtered through four layers of sterile gauze. The pH of each sample was measured on-site using a portable pH meter, and only those within the physiological range (5.8–7.0) were retained. Filtered samples were divided into 5 mL aliquots, flash-frozen in liquid nitrogen, and stored at −80 °C for bacterial and metabolomic analyses (16).

### 2.4 Determination of rumen bacteria community

Bacterial genomic DNA was extracted using the E. Z. N. A. Soil DNA Kit (Omega Bio-Tek, Norcross, GA, United States) according to the manufacturer’s instructions. Before DNA extraction, rumen fluid samples stored at −80 °C were thawed on ice. Approximately 1 mL of each sample was then centrifuged at 12,000 × g for 10 min at 4 °C to pellet the bacterial biomass. The supernatant was discarded, and the bacterial pellet was immediately subjected to DNA extraction. The quantity and quality of the extracted DNA were assessed using the ND-1000 spectrophotometer (NanoDrop, Wilmington, DE, United States) and confirmed via electrophoresis on a 1% agarose gel. The DNA samples were subsequently stored at −80 °C for downstream sequencing analysis. The V3-V4 hypervariable region of the bacterial 16S rRNA gene was amplified using universal primers 338F (5’-ACTCCTACGGAGGCAGG-3′) and 806R (5’-GGACTACHVGGGTWTCTAAT-3′). PCR amplification was performed on an ABI GeneAmp 9,700 thermal cycler (Applied Biosystems, CA, United States) as described by Jiang et al. (17). The integrity of PCR products was verified, followed by purification and quantification. The purified PCR products were pooled in equimolar ratios based on their quantified concentrations. The 16S rRNA gene amplicons were sequenced on the Illumina HiSeq 2,500 platform (Illumina Inc., San Diego, CA, United States). Raw sequencing reads were quality-filtered based on length and quality scores. High-quality reads were then processed to generate effective tags for downstream analysis (18). The raw sequencing data have been deposited in the NCBI Sequence Read Archive under accession numbers PRJNA877011 and PRJNA877811 (Accession Number: PRJNA877011, PRJNA877811).

During the processing of raw 16S rRNA gene sequencing reads, quality filtering and read merging were conducted using Fastp (v0.20.0) and FLASH (v1.2.7), respectively. Operational taxonomic units (OTUs) were clustered using UPARSE (v7.1) at a 97% sequence similarity threshold (19). Taxonomic classification and annotation of OTUs were performed using the SILVA reference database (release 132) and a naive Bayes classifier implemented in QIIME2, with a confidence threshold of 70%. To assess differences in bacterial community abundance between groups, species-level abundance data were compared using the Metastats method based on a t-test.

### 2.5 Determination of rumen fluid metabolites

After thawing, 100 μL of each rumen fluid sample was mixed with 800 μL of acetonitrile:methanol (1:1, v/v) containing internal standards (0.02 mg/mL L-2-chlorophenylalanine, etc.), vortexed for 30 s, and sonicated at 5 °C for 30 min. The mixture was then incubated at −20 °C for 30 min to precipitate proteins, followed by centrifugation at 13,000 × g for 15 min at 4 °C. The resulting supernatant was dried under a stream of nitrogen gas. The residue was reconstituted in 100 μL of acetonitrile:water (1:1, v/v), sonicated again (5 min at 5 °C), centrifuged, and the final supernatant was transferred to vials for subsequent LC–MS/MS analysis. LC–MS analysis was conducted on a UHPLC-Q Exactive HF-X system (Thermo Fisher Scientific, Waltham, MA, United States) equipped with an ACQUITY HSS T3 column (100 mm × 2.1 mm i.d., 1.8 μm; Waters, USA) at Majorbio Bio-Pharm Technology Co. Ltd. (Shanghai, China). The mobile phases consisted of 0.1% formic acid in water:acetonitrile (2:98, v/v) (solvent A) and 0.1% formic acid in acetonitrile (solvent B). The flow rate was set to 0.40 mL/min, the column temperature was maintained at 40 °C, and the injection volume was 5 μL.

The raw LC–MS data were processed using Progenesis QI software to generate a data matrix. Metabolites exhibiting a relative standard deviation (RSD) greater than 30% in quality control samples were excluded to ensure data reliability. The remaining metabolites were annotated by matching to entries in the Human Metabolome Database (HMDB). The LC–MS data underwent baseline correction, peak alignment, and multivariate statistical analysis using Progenesis QI software (v3.0, Waters Corporation, Milford, MA, United States). Principal component analysis (PCA) and orthogonal partial least squares discriminant analysis (OPLS-DA) were performed using the “ropls” R package (version 1.6.2), with 7-fold cross-validation employed to assess model stability. Metabolites with a variable importance in projection (VIP) score >1 and *p*-value <0.05 (Student’s t-test) were considered significantly different. Significantly altered metabolic pathways were visualized using iPath3 (20).

### 2.6 Statistical analysis

Experimental data were organized and processed using Microsoft Excel. Statistical analyses were performed using one-way analysis of variance (ANOVA) in SPSS 24.0 (IBM, Armonk, NY, United States) based on the following model: *Y_ij_* = *μ* + *T_i_* + *ϵ_ij_*, where *Y_ij_* is the observed value for the *j*-th cow in the *i*-th treatment group; *μ* is the overall mean; *Ti* represents the fixed effect of the *i*-th dietary treatment (FCP, HCP, or ICP); and *ϵ_ij_* is the random error term, assumed to be independently and normally distributed. *Post hoc* multiple comparisons between groups were conducted using Tukey’s honestly significant difference test. A *p*-value<0.05 was considered statistically significant.

## 3 Results

### 3.1 Dry matter intake and lactation performance of the entire experimental period

No significant effects of dietary treatment were observed on dry matter intake (*p* = 0.612), milk yield (*p* = 0.469), milk fat content (*p* = 0.605), milk protein content (*p* = 0.533), or lactose concentration (*p* = 0.403), as shown in Table 3.

**Table 3:** Effect of dietary CP content on lactation performance of dairy cows.

Items	HCP	ICP	FCP	SEM	*p-*value
Dry matter intake(kg/d)	18.66	18.86	18.62	0.203	0.612
Milk yield(kg/d)	24.83	25.70	26.57	1.395	0.469
Milk fat concentration (%)	3.20	3.33	3.15	0.177	0.605
Milk protein concentration (%)	3.30	3.38	3.27	0.079	0.533
Lactose concentration	5.30	5.26	5.33	0.041	0.403

Means within rows with different superscripts are significantly different (*p* < 0.05).

### 3.2 Urea nitrogen in milk, plasma, and urine of final fluctuation cycle

To assess the temporal effects of CP fluctuation, nitrogen partitioning in plasma, milk, and urine was analyzed over a complete 4-day cycle (Table 4). At the beginning of the high CP phase (D1), PUN in the FCP group was significantly lower (10.34 mg/dL) than in the HCP group (14.55 mg/dL; *p* = 0.024), while no significant differences in MUN or UUN were observed among the groups at this time point. As the high CP phase progressed (D2), MUN increased markedly in the FCP group to 16.99 mg/dL, reaching a level comparable to HCP (16.69 mg/dL) but significantly higher than ICP (13.58 mg/dL; *p* < 0.001). PUN also rose in the FCP group (14.84 mg/dL) but did not differ significantly from either HCP or ICP. UUN reached 422.10 mg/dL in FCP, higher than ICP (366.36 mg/dL), although the difference was not statistically significant. During the transition to the low CP phase (D3), PUN and UUN in the FCP group peaked at 14.25 mg/dL and 483.41 mg/dL, respectively, values significantly higher than those in HCP (PUN: 12.32 mg/dL; UUN: 340.20 mg/dL) and ICP (PUN: 10.40 mg/dL; UUN: 266.70 mg/dL; *p* < 0.05). MUN remained elevated in FCP (17.41 mg/dL), significantly higher than in ICP (14.38 mg/dL; *p* < 0.001). By the end of the cycle (D4), a sharp decline in PUN was observed in FCP group (9.87 mg/dL), significantly lower in than HCP (13.89 mg/dL; *p* < 0.001). MUN and UUN also decreased in the FCP group to 15.71 mg/dL and 294.30 mg/dL, respectively, although these decreases were not statistically significant.

**Table 4:** Effect of fluctuating dietary crude protein (CP) content on nitrogen utilization of Urea nitrogen in milk, plasma, and urine of the final fluctuation cycle.

Items	Date	HCP	ICP	FCP	SEM	*p-*value
Milk urea nitrogen (mg/dL)	Day 1	14.61	12.01	13.08	1.092	0.079
	Day 2	16.69^a^	13.58^b^	16.99^a^	0.869	<0.001
	Day 3	18.65^a^	14.38^b^	17.41^a^	0.945	<0.001
	Day 4	17.34	15.59	15.71	1.011	0.179
Plasma urea nitrogen (mg/dL)	Day 1	14.55^a^	12.21^ab^	10.34^b^	1.411	0.024
	Day 2	13.62	12.17	14.84	1.301	0.146
	Day 3	12.32^b^	10.40^c^	14.25^a^	0.922	0.002
	Day 4	13.89^a^	10.66^b^	9.87^b^	0.632	<0.001
Urine urea nitrogen (mg/dL)	Day 1	490.12	363.98	336.38	72.350	0.101
	Day 2	493.84	366.36	422.10	67.135	0.188
	Day 3	340.20^b^	266.70^b^	483.41^a^	65.998	0.011
	Day 4	399.74	243.26	294.30	64.881	0.070

Means within rows with different superscripts are significantly different (*p* < 0.05). Day 1-Day 4 represent the 1st to 4th days of the last test cycle. Day 1-Day 2 of the FCP group were high CP diet (18.05% CP, FCP-HCP), and D3-D4 were fed low CP diet (14.04% CP, FCP-LCP). ^a,b^Means for each trait with different superscripts differ significantly at *p* < 0.05.

### 3.3 Rumen bacterial composition

**Figure 1:**
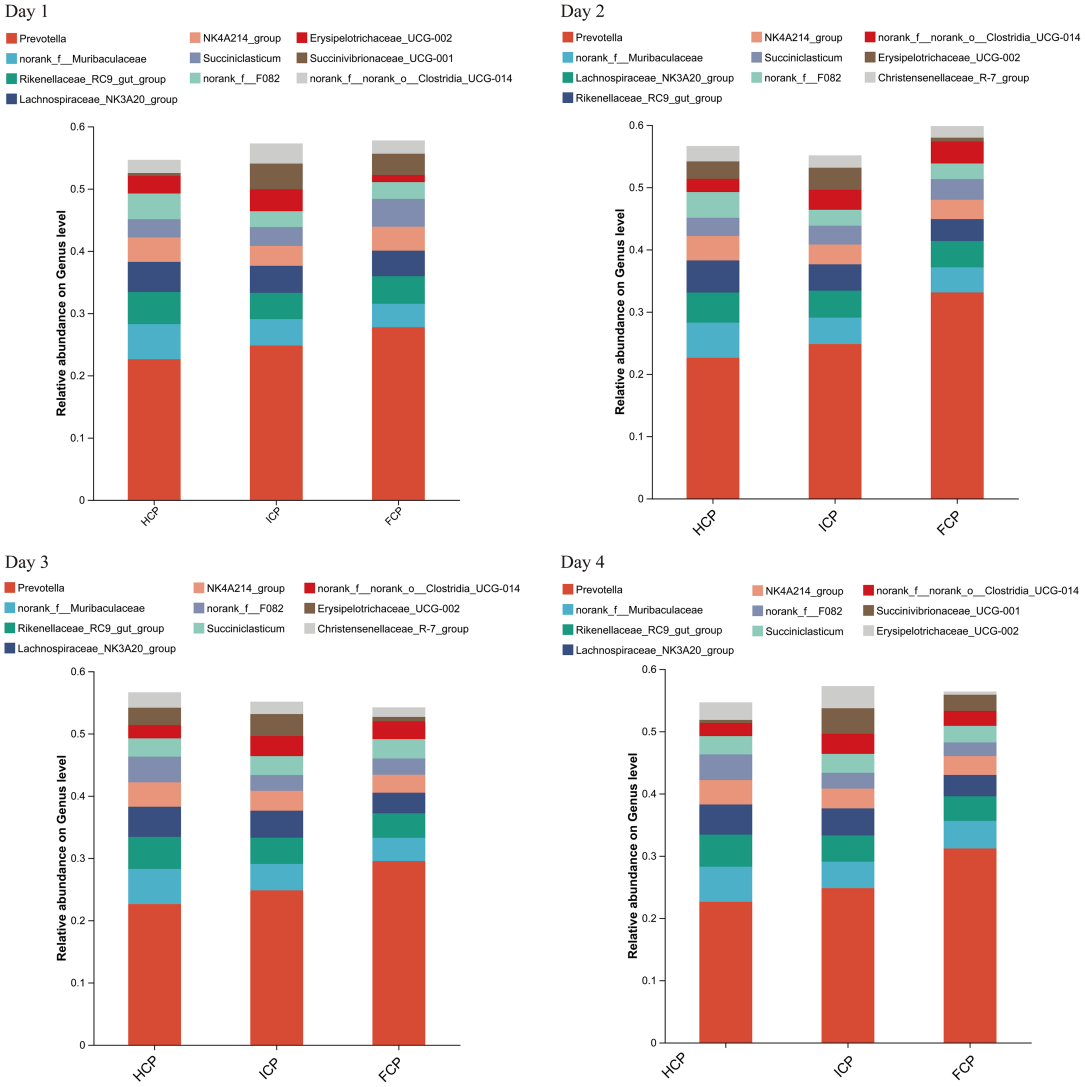
Bar chart of the top ten genera in abundance of rumen fluid among HCP, ICP, and FCP at the genus level over a four-day period of the final fluctuation cycle.

**Figure 2:**
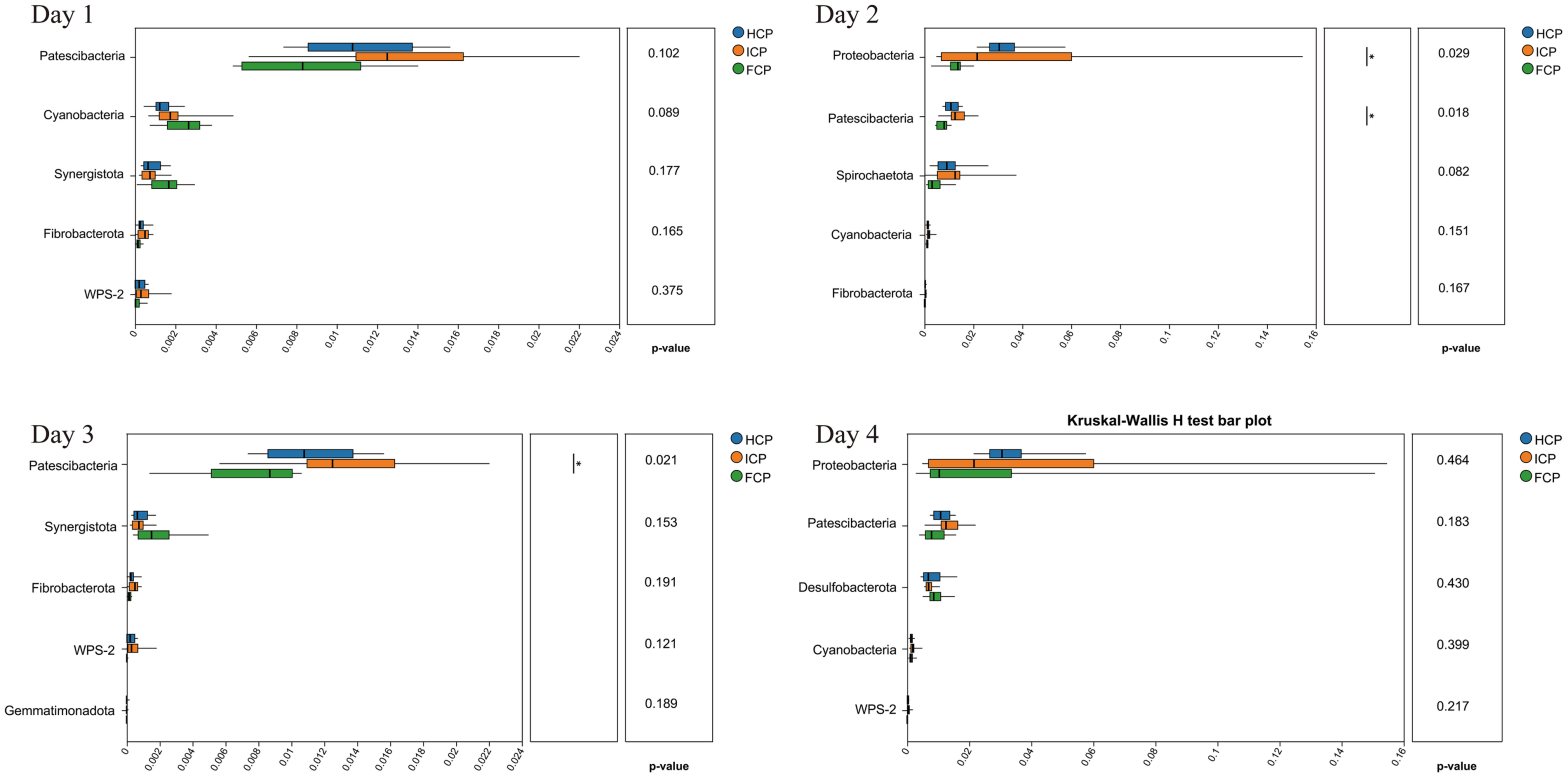
16S rRNA differential analysis of rumen fluid among HCP, ICP, and FCP at the phylum over a four-day period the final fluctuation cycle.

The OTU annotation results at the genus level identified seven persistent core taxa (*Prevotella, norank_f__Muribaculaceae, Rikenellaceae_RC9_gut_group, Lachnospiraceae_NK3A20_group, NK4A214_group, Succiniclasticum, and norank_f__norank_o__Clostridia_UCG-014*) consistently ranked among the top 10 genera across all 4 days, indicating stable presence irrespective of dietary phase (Figure 1). At the phylum level, a total of 22 phyla were detected on Day 1, increasing slightly to 23 phyla on both Day 2 and Day 3, and returning to 22 on Day 4. The six most abundant phyla throughout the four-day fluctuating period were Bacteroidota, Firmicutes, Proteobacteria, Actinobacteriota, Spirochaetota, and Patescibacteria, collectively comprising the majority of the rumen bacterial community. On Day 2, the relative abundances of Proteobacteria and Patescibacteria in the FCP group were significantly lower than those in the HCP and ICP groups (*p* < 0.05). On Day 3, the relative abundance of Patescibacteria remained significantly lower in the FCP group compared to both HCP and ICP groups (*p* < 0.05). Additionally, a trend toward decreased abundance of Spirochaetota was observed in the FCP group on Day 2 compared to HCP and ICP groups (Figure 2).

**Figure 3:**
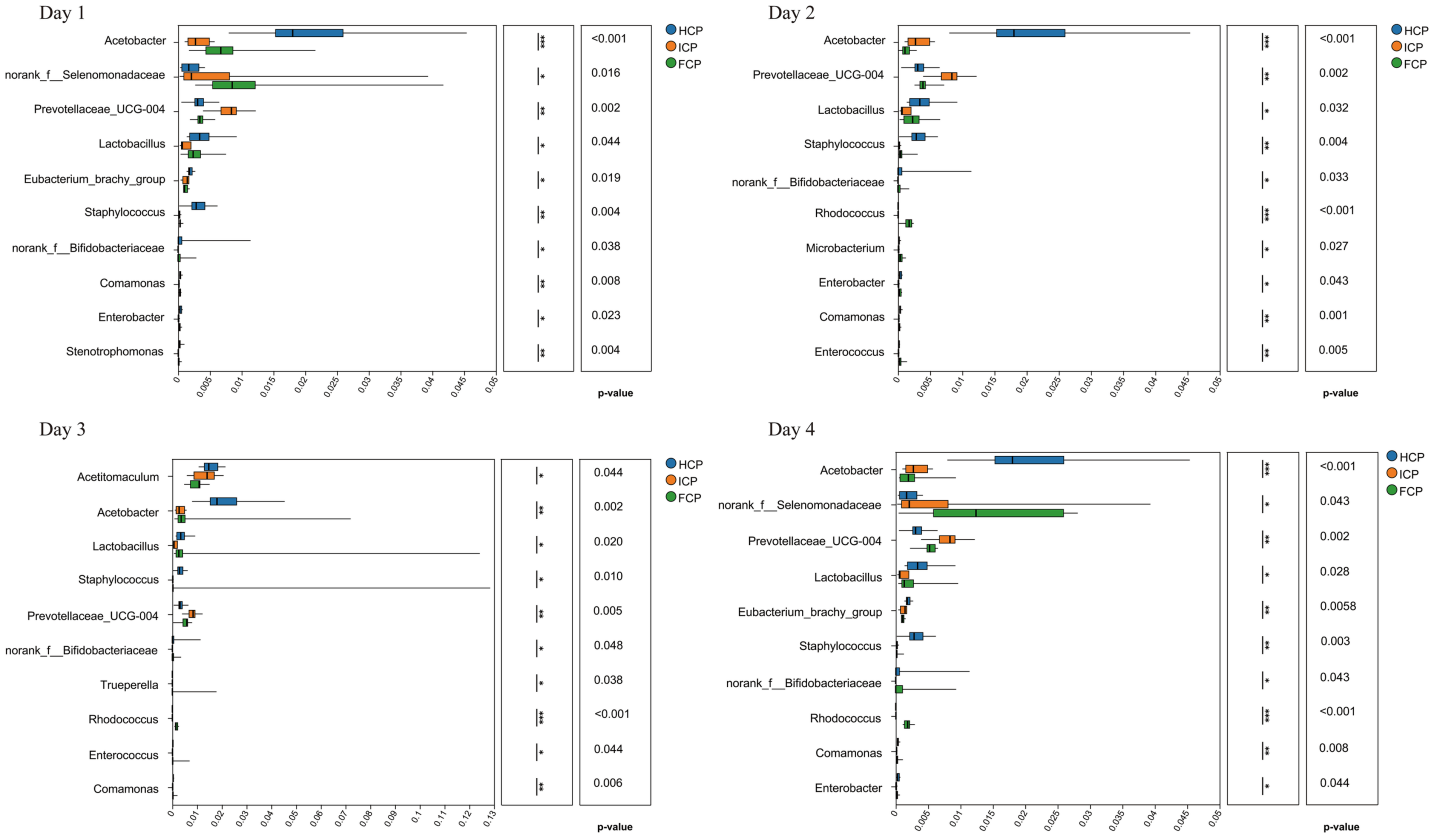
16S rRNA differential analysis of rumen fluid among HCP, ICP, and FCP at the genus level over a four-day period the final fluctuation cycle.

The top 10 genera with significant differences in abundance during the fluctuation period included *Acetobacter*, *norank_f__Selenomonadaceae*, *Prevotellaceae_UCG-004*, *Lactobacillus*, *Eubacterium_brachy_group*, *Staphylococcus*, *norank_f__Bifidobacteriaceae*, and *Comamonas*, among others (*p* < 0.05) (Figure 3). In Day 1, nine genera exhibited divergent abundance (*p* < 0.05), including *norank_f__Selenomonadaceae*, *Prevotellaceae_UCG-004*, and *Lactobacillus*, with FCP suppressing *Enterobacter* (HCP/ICP > FCP) and *Stenotrophomonas* (ICP > FCP/HCP). Day 2 highlighted reduced abundance of fiber-degrading *Rhodococcus* in FCP versus HCP/ICP, while *Enterococcus* was elevated in ICP. By Day 3, *Acetobacter* (acid-producing) and *Trueperella* (opportunistic) emerged as differentially abundant, with FCP consistently reducing *Prevotellaceae_UCG-004* and *Comamonas*. Day 4 confirmed sustained effects: *Lactobacillus* remained lower in FCP, while *Rhodococcus* and *norank_f__Bifidobacteriaceae* diverged significantly across diets.

### 3.4 Rumen fluid metabolites

**Figure 4:**
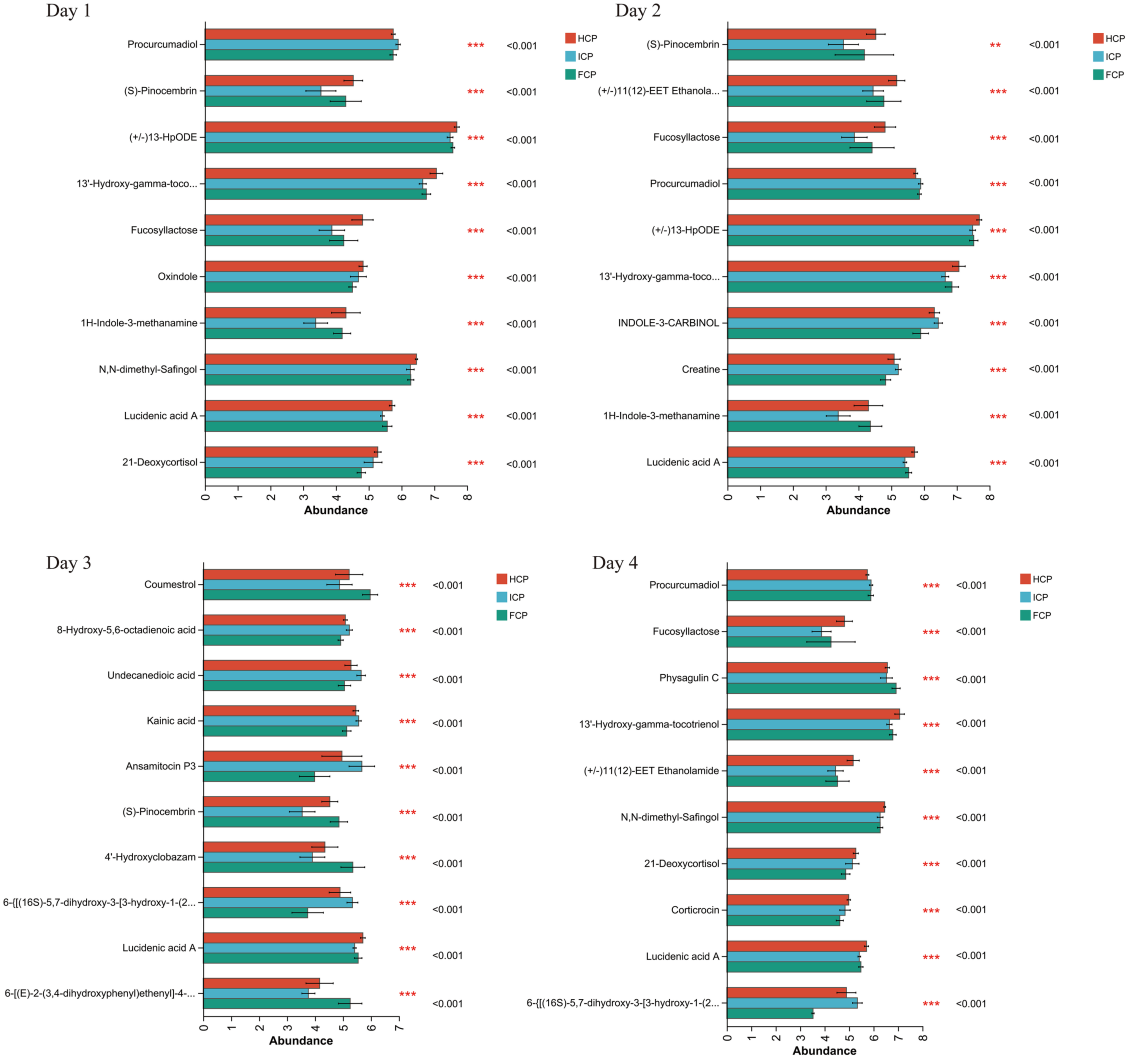
Metabolome differential in rumen fluid among HCP, ICP, and FCP over a four-day period the final fluctuation cycle.

Differential metabolites among treatment groups were identified based on the criteria of variable importance in projection (VIP) > 1, *p*-value < 0.05, and |Log_2_FC| > 1. Over the four-day period, comparative analyses revealed numerous metabolites that differed significantly in abundance among the three dietary groups. The top discriminating metabolites identified during the fluctuation cycle are shown in Figure 4.

To explore the biological relevance of these differential metabolites, KEGG pathway topology analysis was performed, and an enrichment table of significantly affected pathways was generated. On Day 1, comparisons between the FCP and HCP groups revealed metabolic disturbances in several pathways, including benzoxazinoid biosynthesis, ubiquinone and other terpenoid-quinone biosynthesis, and bisphenol degradation (Table 5). On Day 2, significant alterations were observed in amino acid metabolism, particularly involving alanine, aspartate, glutamate, glycine, serine, and threonine. On Day 3, the affected metabolic pathways included glutathione metabolism, arginine biosynthesis, and tyrosine metabolism. On Day 4, metabolic disturbances were again noted in benzoxazinoid biosynthesis, along with steroid hormone biosynthesis.

**Table 5:** Enrichment of KEGG differential pathways in the rumen fluid of dairy cows in the final fluctuation cycle between the FCP and HCP.

Date	ID	Pathway	*p-*value	Compounds (KEGG ID)	Metabolite
Day 1	map00402	Benzoxazinoid biosynthesis	0.024	C12312	Oxindole
	map00130	Ubiquinone and other terpenoid-quinone biosynthesis	0.007	C14155 C00156	Gamma-Tocotrienol, P-Salicylic acid
	map00363	Bisphenol degradation	0.011	C00156	P-Salicylic acid
Day 2	map00250	Alanine, aspartate and glutamate metabolism	0.042	C00049	L-Aspartic acid
	map00260	Glycine, serine and threonine metabolism	0.002	C00300 C00049	Creatine, L-Aspartic acid
Day 3	map00480	Glutathione metabolism	0.036	C03740 C00051	5-L-Glutamyl-L-alanine, Glutathione
	map00220	Arginine biosynthesis	0.014	C01250 C00624	Acetyl-L-glutamate 5-semialdehyde, N-Acetyl-L-glutamic acid
	map00350	Tyrosine metabolism	0.011	C05588 C00788 C00822	Metanephrine (−)-Epinephrine Dopaquinone
Day 4	map00402	Benzoxazinoid biosynthesis	0.035	C12312	Oxindole
	map00140	Steroid hormone biosynthesis	0.040	C05478 C05497	3a,21-Dihydroxy-5b-pregnane-11,20-dione, 21-Deoxycortisol

Day 1-Day 4 represent the 1st to 4th days of the last test cycle. Day 1-Day 2 of the FCP group were high CP diet (18.05% CP, FCP-HCP), and Day 3-Day 4 were fed low CP diet (14.04% CP, FCP-LCP).

In the comparison between FCP and ICP groups, Day 1 showed disturbances in several metabolic pathways, including alanine, aspartate and glutamate metabolism; pantothenate and CoA biosynthesis; caffeine metabolism; toluene degradation; histidine metabolism; purine metabolism; drug metabolism—other enzymes; and arginine biosynthesis (Table 6). On Day 2, the affected metabolic pathways included linoleic acid metabolism, pyrimidine metabolism, and benzoxazinoid biosynthesis. On Day 3, significant alterations were observed in metabolic pathways such as arginine biosynthesis, beta-alanine metabolism, phenylpropanoid biosynthesis, and tyrosine metabolism. On Day 4, the metabolic changes were primarily enriched in flavonoid biosynthesis. Notably, N-acetyl-L-glutamic acid was significantly enriched on Day 3 within the arginine biosynthesis pathway in both HCP vs. FCP and ICP vs. FCP comparisons.

**Table 6:** Enrichment of KEGG differential pathways in rumen fluid of dairy cows in the final fluctuation cycle between the FCP and ICP.

Date	ID	Pathway	*p-*value	Compounds (KEGG ID)	Metabolite
Day 1	map00250	Alanine, aspartate and glutamate metabolism	0.020	C01042 C00049	N-acetylaspartate, L-Aspartic acid
	map00770	Pantothenate and CoA biosynthesis	0.018	C00864 C00049	Pantothenic Acid, L-Aspartic acid
	map00232	Caffeine metabolism	0.009	C01762 C00385	Xanthosine, Xanthine
	map00623	Toluene degradation	0.039	C00261 C00633	Benzaldehyde, 4-Hydroxy-benzaldehyde
	map00340	Histidine metabolism	0.026	C00785 C00049	Urocanic acid, L-Aspartic acid
	map00230	Purine metabolism	0.000	C00262 C05512 C00366 C01762 C00385	Hypoxanthine, Deoxyinosine, Uric acid, Xanthosine, Xanthine
	map00983	Drug metabolism—other enzymes	0.035	C07446 C16635	Isonicotinic acid, 5’-Deoxy-5-fluorocytidine
	map00220	Arginine biosynthesis	0.014	C00624 C00049	N-Acetyl-L-glutamic acid, L-Aspartic acid
Day 2	map00591	Linoleic acid metabolism	0.037	C14831	8(R)-Hydroperoxylinoleic acid
	map00240	Pyrimidine metabolism	0.011	C00178 C00106	Thymine, Uracil
	map00402	Benzoxazinoid biosynthesis	0.026	C00463	Indole
Day 3	map00220	Arginine biosynthesis	0.025	C01250 C00624	N-Acetyl-L-glutamate 5-semialdehyde, N-Acetyl-L-glutamic acid
	map00410	Beta-Alanine metabolism	0.029	C00555 C00864	4-Amino-butyraldehyde, Pantothenic Acid
	map00940	Phenylpropanoid biosynthesis	0.026	C18069 C02325 C05838	N1, N5, N10-Tricoumaroyl spermidine, Sinapyl alcohol, 2-Hydroxycinnamic acid
	map00350	Tyrosine metabolism	0.024	C05588 C00788 C00822	Metanephrine, (−)-Epinephrine, Dopaquinone
Day 4	map00941	Flavonoid biosynthesis	0.031	C09827 C05904	(S)-Pinocembrin, Pelargonidin

Day 1-Day 4 represent the 1st to 4th days of the last test cycle. Day 1-Day 2 of the FCP group were high CP diet (18.05% CP, FCP-HCP), and Day 3-Day 4 were fed low CP diet (14.04% CP, FCP-LCP).

## 4 Discussion

Previous studies have shown that short-term fluctuations in dietary crude protein (CP) levels do not necessarily impair lactation performance in dairy cows. Tebbe et al. (15) found that cows fed a fluctuating CP diet (every 24 h) exhibited nitrogen utilization, milk protein content, and fat-corrected milk yield comparable to those fed a stable CP diet. Similarly, Rauch et al.’s study reported that cows fed a diet with varying levels of CP, ranging from low to high, exhibited similar performance to those fed a stable CP diet. Furthermore, milk yield, milk composition, body weight, nitrogen efficiency, and feed utilization were not adversely affected by the fluctuation in CP levels (21). Broderick also reported no significant changes in lactation performance when dietary CP levels fluctuated between 16 and 18% (22). Our findings, consistent with previous studies, confirm the resilience of lactation performance to CP fluctuations and suggest that this stability may reflect evolved metabolic adaptations in ruminants. With dry matter intake held constant, the total nitrogen intake of the FCP group (16.05% CP) during the fluctuation cycle was lower than that of the HCP group (18.05% CP). These observations likely reflect evolved metabolic adaptations and host-microbiome interactions that buffer short-term protein fluctuations to maintain nitrogen homeostasis. Improved nitrogen retention may be attributed to increased ruminal urea concentrations during the low CP phase, which could enhance nitrogen utilization efficiency (23). Although the nitrogen intake was identical between the ICP and HCP groups, their lactation performance remained similar. This may be due to the rumen microbiome’s ability to dynamically regulate urease activity (24) and recycle endogenous nitrogen via the ornithine-urea cycle may buffer short-term CP variations (25).

To elucidate the physiological mechanisms underlying the dynamic response to fluctuating dietary CP, we analyzed the temporal changes in nitrogen partitioning across plasma, milk, and urine over a complete 4-day cycle. At the onset of the high CP phase, the FCP group exhibited significantly lower PUN than the HCP group. This may reflect a hepatic adaptation that downregulates ureagenesis to conserve nitrogen and replenish the ruminal NH₃–N pool, compensating for its depletion under prior low CP intake (26, 27). Such a mechanism has been proposed as a nitrogen-sparing strategy during transient protein deficiency, enhancing the subsequent availability of nitrogen for productive purposes, such as milk protein synthesis. As the high CP phase progressed, PUN and MUN levels rose in the FCP group, approaching values similar to the HCP group. However, UUN did not exhibit a parallel increase until the Day 3. This temporal lag suggests that urinary nitrogen excretion does not respond immediately to dietary CP changes, possibly due to transient shifts in hepatic nitrogen handling and enhanced urea recycling to the rumen (28). The observed UUN peak on Day 1 of the low CP phase indicates that urinary nitrogen losses are influenced not only by current intake but also by prior nitrogen loads and the kinetics of nitrogen redistribution.

The sustained elevation of MUN during the early low CP phase likely reflects a systemic urea pool carried over from the preceding high CP period. Given that MUN is largely equilibrated with blood urea, it may serve as a delayed indicator of whole-body nitrogen status rather than an immediate marker of mammary nitrogen utilization (21). Moreover, the absence of a significant decrease in UUN at the onset of the low CP phase underscores the complexity of nitrogen excretion regulation and the buffering capacity of the ruminant system. Urea recycling to the rumen likely plays a key role in moderating the impact of CP fluctuations. Studies have shown that bacterial urease activity and epithelial urea transport are upregulated under conditions of reduced nitrogen supply, enhancing the capture of recycled urea for microbial protein synthesis (24, 25). This mechanism partially compensates for reduced dietary nitrogen, explaining the transient stability observed in nitrogen excretion patterns. However, this bacterial adaptation likely requires time for full activation, contributing to the delayed decline in UUN following CP reduction. These findings demonstrate that ruminants dynamically coordinate hepatic, renal, and ruminal urea metabolism to adapt to short-term CP fluctuations, promoting nitrogen allocation toward productive functions rather than excretion.

Rumen bacteria play a critical role in feed degradation and nutrient supply (29). The composition and function of the rumen bacterial community are strongly influenced by diet, which in turn affects feed efficiency and nitrogen metabolism in ruminants (30, 31). Alterations in dietary CP levels can modulate the rumen microbiota’s diversity and function, thereby influencing NUE and overall nitrogen homeostasis. In this study, 16S rRNA gene sequencing revealed that fluctuating dietary CP levels induced shifts in specific bacterial taxa without significantly affecting overall alpha diversity. This finding suggests the presence of a resilient core bacterial community capable of maintaining functional stability despite short-term fluctuations in protein supply. The observed bacterial shifts likely contribute to the dynamic nitrogen partitioning and recycling patterns reflected in our nitrogen metabolism data (10). At the phylum level, Proteobacteria—many members of which possess nitrogen-fixing and ureolytic capabilities—showed a significant decrease on Day 2 in the FCP group compared to the HCP and ICP groups. This decrease parallels the reduced nitrogen intake observed in the FCP group and may indicate bacterial adaptation to lower nitrogen availability, potentially conserving nitrogen by downregulating populations less essential under nitrogen-limited conditions. Considering the role of Proteobacteria in nitrogen fixation and ammonia assimilation, their reduced abundance may represent a bacterial strategy to optimize nitrogen cycling efficiency during low-CP phases.

At the genus level, *Prevotella* remained the dominant genus with stable abundance across all treatments, consistent with its recognized role in degrading carbohydrates and proteins (32, 33). Species like *Prevotella ruminicola* specialize in oligopeptide degradation, facilitating nitrogen recycling through the breakdown of peptides into amino acids available for bacterial protein synthesis. The stability of *Prevotella* populations despite dietary CP fluctuations suggests that key protein-degrading functions remain robust, ensuring continuous nitrogen availability for bacterial growth and fermentation. This functional redundancy may underpin the maintenance of rumen bacterial protein synthesis and host nitrogen retention observed in the FCP group. Interestingly, *Prevotellaceae_UCG-004*, an unclassified genus-level cluster within the *Prevotellaceae* family, showed a modest but consistent increase in the HCP group. This differential response may reflect niche differentiation within the Prevotellaceae, where *Prevotellaceae_UCG-004* exhibits specialized metabolic traits optimized for higher dietary protein conditions (24). Such specialization could contribute to more efficient nitrogen metabolism under high CP intake, supporting elevated bacterial protein synthesis and possibly facilitating enhanced nitrogen retention as suggested by our nitrogen partitioning results.

Non-targeted metabolomics further revealed altered metabolic pathways in the FCP group, notably alanine, aspartic acid, glutamic acid metabolism, arginine biosynthesis, and tyrosine metabolism compared to HCP and ICP. L-aspartate, a key amino group donor involved in multiple metabolic pathways including the urea cycle and sugar metabolism, was enriched on Days 1 and 3 of the fluctuating cycle. It participates in *β*-alanine synthesis, precursor for pantothenic acid and coenzyme A biosynthesis (34), pathways also altered in our study. These changes may reflect bacterial polysaccharide storage during nitrogen deficiency and subsequent mobilization when nitrogen is adequate (35). The close synchronization between nitrogen and energy metabolism (36) suggests that dairy cows adjust metabolic allocation in response to dietary CP shifts, alleviating stress from protein fluctuations.

N-Acetyl-L-glutamic acid, an activator of carbamoyl phosphate synthase I that facilitates arginine synthesis, was also implicated in the metabolic changes observed. Since animals are unable to synthesize arginine via the ornithine-urea cycle due to enzyme constraints, alterations in arginine biosynthesis likely reflect enhanced ruminal nitrogen recycling. Fluctuations in tyrosine metabolism, which is known to influence milk protein synthesis (37), were also detected. Despite these metabolic changes, overall milk protein yield remained stable, likely owing to the complex regulatory mechanisms controlling protein synthesis. The dynamic shifts in bacterial taxa correspond with metabolomic data indicating modifications in amino acid metabolic pathways, including aspartate and arginine biosynthesis, both closely associated with nitrogen recycling and urea cycling in the rumen (38). The enrichment of L-aspartate, a critical nitrogen donor, during the CP fluctuation cycle suggests coordinated metabolic responses between the rumen microbiota and the host to maintain nitrogen homeostasis. Collectively, these bacterial community adjustments and metabolic adaptations contribute to efficient nitrogen utilization, thereby explaining the stable lactation performance despite dietary protein fluctuations.

## 5 Conclusion

In conclusion, this study demonstrates that fluctuations in dietary crude protein content lead to increased concentrations of MUN and UUN, alongside decreased PUN concentrations at different stages. Despite these changes, lactation performance remained unaffected, even as the rumen bacterial community structure and metabolic profiles shifted. These findings shed light on the metabolic mechanisms underpinning ruminal adaptation to protein fluctuations and offer a theoretical basis for developing dynamic dietary strategies aimed at optimizing nitrogen metabolism in dairy cows.

## Data Availability

The datasets presented in this study can be found in online repositories. The names of the repository/repositories and accession number(s) can be found at: https://www.ncbi.nlm.nih.gov/, PRJNA877011; https://www.ncbi.nlm.nih.gov/, PRJNA877811.
